# The role of EMILIN-1 in the osteo/odontogenic differentiation of dental pulp stem cells

**DOI:** 10.1186/s12903-023-02905-3

**Published:** 2023-04-06

**Authors:** Pingmeng Deng, Jing Huang, Qixuan Zhang, Yuejia Li, Jie Li

**Affiliations:** 1 College of Stomatology, Chongqing Medical University, 426# Songshibei Road, Yubei District, Chongqing, 401147 People’s Republic of China; 2 Chongqing Key Laboratory of Oral Diseases and Biomedical Sciences, Chongqing, People’s Republic of China; 3 Chongqing Municipal Key Laboratory of Oral Biomedical Engineering of Higher Education, Chongqing, People’s Republic of China

**Keywords:** Elastin microfibril interface-located protein-1, Human dental pulp stem cells, Osteo/Odontogenic differentiation, Tooth regeneration, Bone regeneration

## Abstract

**Background:**

Human dental pulp stem cells (hDPSCs) may be the best choice for self-repair and regeneration of teeth and maxillofacial bone tissue due to their homogeneous tissue origin, high proliferation and differentiation rates, and no obvious ethical restrictions. Recently, several studies have shown that extracellular matrix (ECM) proteins can effectively regulate the proliferation and differentiation fate of mesenchymal stem cells (MSCs). However, the role of elastin microfibril interface-located protein-1 (EMILIN-1), a new ECM glycoprotein, in osteo/odontogenic differentiation of hDPSCs has not been reported. The aim of this study was to explore the effect of EMILIN-1 during osteo/odontogenic differentiation of hDPSCs.

**Methods:**

hDPSCs were cultured in osteo/odontogenic induction medium. qPCR and Western blot analysis were performed to detect osteo/odonto-specific genes/proteins expression as well as the expression of EMILIN-1. After knockdown of *Emilin-1* in hDPSCs with small interfering RNA and exogenous addition of recombinant human EMILIN-1 protein (rhEMILIN-1), Cell Counting Kit-8 assay, alkaline phosphatase staining, alizarin red S staining, qPCR and Western blot were performed to examine the effect of EMILIN-1 on proliferation and osteo/odontogenic differentiation of hDPSCs.

**Results:**

During the osteo/odontogenic induction of hDPSCs, the expression of osteo/odonto-specific genes/proteins increased, as did EMILIN-1 protein levels. More notably, knockdown of *Emilin-1* decreased hDPSCs proliferation and osteo/odontogenic differentiation, whereas exogenous addition of rhEMILIN-1 increased them.

**Conclusions:**

These findings suggested that EMILIN-1 is essential for the osteo/odontogenic differentiation of hDPSCs, which may provide new insights for teeth and bone tissue regeneration.

**Supplementary Information:**

The online version contains supplementary material available at 10.1186/s12903-023-02905-3.

## Background

Defects of teeth and maxillofacial bones caused by caries, periodontal diseases, trauma, tumor, etc. affect people’s health. Traditional restoration methods, such as root canal treatment, crown restoration and dental implants, can only restore their structure, but not their function completely. The loss of tissue bioactivity and the increased brittleness greatly increase the probability of tooth fracture and cracking. Notably, the regeneration mediated by mesenchymal stem cells (MSCs) is expected to restore the structure and function as well as the biological activity of teeth and maxillofacial bone tissue. Compared with human bone marrow mesenchymal stem cells (hBMSCs), human dental pulp stem cells (hDPSCs) have stronger proliferation ability [1], and based on the same tissue origin, hDPSCs have superior feasibility for repair and regeneration of teeth and maxillofacial bone tissue. In addition, hDPSCs have the advantages of high osteo/odontogenic differentiation capacity, easy accessibility and low immunogenicity [1, 2]. Therefore, hDPSCs have become an ideal source of stem cells for the study of tooth and bone repair and regeneration. The dentin matrix and the bone matrix contain a variety of proteins and other molecules known as their extracellular matrix (ECM) components. Studies have shown that MSCs-mediated regeneration of teeth and bone tissue is regulated by ECM proteins in the microenvironment in which the cells survive [3, 4].

ECM contains a large number of proteins, glycans and various cytokines, and studies have shown that ECM components are key determinants of tissue development, homeostasis and disease progression [5]. ECM proteins work as regulators and integrators in the signaling crosstalk between cells and the matrix [6]. Elastin Microfibril Interface Located proteINs (EMILINs) [7] are a novel family of extracellular matrix glycoproteins, including elastin microfibril interface-located protein-1 (EMILIN-1), EMILIN-2, EMILIN-3, EMILIN-4, and EMILIN-5 [8], which are abundantly expressed in elastic-rich tissues such as heart, blood vessels and skin [9], and are closely associated with elastogenesis, development of the cardiovascular system and progression of related diseases, and tumor growth [10–13]. Notably, an association between EMILINs and bone metabolism has been reported. Alvise Schiavinato et al. found that EMILIN-1 and EMILIN-2 are deposited in the skeletal system, such as the extracellular matrix of bone and cartilage [14]. Mitsuhito Doi et al. concluded that EMILIN-5 plays an important role in skeletal development because EMILIN-5 is highly expressed in perichondrium cells of developing limbs of embryonic mice [8]. Among them, EMILIN-1 is the first and most characterized member of the EMILIN family. EMILIN-1 is strongly expressed in mesenchymal condensations such as limb buds and branchial arches in embryonic mice, suggesting that EMILIN-1 may be involved in bone and tooth morphogenesis and differentiation [15]. Our previous study found that EMILIN-1 was detected in human dentin for the first time [16]. In addition, Chun Yuan et al. found that EMILIN-1 contributed to fracture healing [17]. Therefore, EMILIN-1 may play an important role in the formation and regeneration of tooth and bone tissue.

Taken together, EMILIN-1 may play a role in the repair and regeneration of teeth and bone tissue, as well as in the osteo/odontogenic differentiation of hDPSCs. However, the effect of EMILIN-1 on the osteo/odontogenic differentiation of hDPSCs has not been reported to date. In this study, the effect of EMILIN-1 on osteo/odontogenic differentiation in hDPSCs was explored by knocking down EMILIN-1 in hDPSCs using small interfering RNA (siRNA) and by adding recombinant human EMILIN-1 protein (rhEMILIN-1).

## Methods

### Isolation and culture of primary hDPSCs

The third molars of patients aged 16–22 who were treated in the Department of Maxillofacial Surgery, Stomatological Hospital of Chongqing Medical University were obtained. The collection of teeth was approved by the Ethics Committee of Chongqing Medical University (CQHS-REC-2021 (LSNo 40)) and written informed consent was obtained from the participants. Simply put, the pulp was removed from the pulp cavity, cut into cubes of about 1 mm^3^ and then digested in 3 mg/mL type I collagenase (Sigma, St. Louis, MO, USA) for 35–40 min at 37 °C. The digested tissue was evenly coated on the bottom of a T 25 culture bottle (Jet Bio, Guangzhou, China), which was cultured in a cell incubator with saturated humidity and 5% CO_2_ at 37 °C. After 8–12 h, the growth medium (GM) consisting of 90% alpha-Minimum Essential Medium (α-MEM; HyClone, Logan, UT, USA), 10% fetal bovine serum (FBS; Biological Industries, Israel) and 1% Penicillin–Streptomycin 100 × solution (HyClone, Logan, UT, USA) was gently added to the culture bottle to soak the tissue blocks evenly. When cell proliferation reached about 80%-90%, the cells were subcultured with trypsin (Mengbio, Chongqing, China) in a ratio of 1:3. All hDPSCs used in this experiment were from the third passage to the fifth passage.

### Flow cytometry analysis

To confirm that the obtained hDPSCs were MSCs, flow cytometry was performed to detect molecular markers on the cell surface. In simple terms, hDPSCs were collected by trypsin digestion and then respectively incubated with 10 µL of the following antibodies (CD14-FITC, CD19-FITC, CD45-FITC, CD90-FITC, and CD105-FITC (BD Pharmingen, San Diego, CA, USA) in a light-protected centrifuge tube (Jet Bio, Guangzhou, China) for 30 min at room temperature. Then the cells were repeatedly washed with phosphate buffered saline (PBS; Solarbio, Beijing, China) containing 2% FBS to remove remaining antibodies. Subsequently, the expression of cell surface molecular markers were detected by BD flow cytometry (BD Biosciences, San Jose, CA, USA).

### Colony-forming assay

The hDPSCs were grown with GM for 10 days after being inoculated on a 6 cm diameter culture dish (Jet Bio, Guangzhou, China) at 500 cells/well. After the culture medium was discarded, the cells was carefully washed 3 times with PBS and then fixed with 4% paraformaldehyde fix solution (PFA, Beyotime, Shanghai, China) for 15 min. After washing with distilled water to remove residual PFA, the cells were stained for 10 min with crystal violet staining solution (Beyotime, Shanghai, China). The cells were then washed sufficiently with distilled water, air dried, scanned in the Epson Perfection V330 scanner (Epson Co., Ltd., Shanghai, China) and photographed under the inverted light microscope (Leica, Wetzlar, Germany).

### Endogenous *Emilin-1* silencing by siRNA

The hDPSCs were inoculated in 6-well plates at 2 × 10^5^ cells/well and cultured in the GM until the cells aggregated to about 60%. According to the instructions, Lipofectamine™ 3000 (Thermo Fisher Scientific, Waltham, MA, USA) was diluted with Opti-MEM reduced serum medium (GIBCO BRL, Grand Island, NY, USA) to prepare the mixture, which was used to incubate directly with hDPSCs for 10 min at room temperature. Next, siRNA (non-specific control siRNA (siNC), si*Emilin-1-a*, si*Emilin-1-b*, and si*Emilin-1-c*) was diluted with Opti-MEM reduced serum medium, and then the diluted siRNA was incubated with hDPSCs at 37 °C for 24 h. hDPSCs without siRNA transfection were used as blank control (Ctrl). At this point, the transfection efficiency of the hDPSCs was assayed. In the subsequent experiments, hDPSCs were transfected for 24 h, and the transfection reagent was immediately replaced with the corresponding medium for processing. siRNA was designed by Sangon Biotech (Shanghai) Co., Ltd. (Shanghai, China), sequences for endogenous *Emilin-1* silencing by siRNA are listed in Table 1.

**Table 1:** Sense and antisense sequences for *Emilin-1* silencing by siRNA

Genes	Primers	Sequences (5'-3')
*Emilin-1-a*	Forward Reverse	GCAACCAAGGACCGUAUCAUUTT AAUGAUACGGUCCUUGGUUGCTT
*Emilin-1-b*	Forward Reverse	GAGGCUAUUAUGAUCCAGAGATT UCUCUGGAUCAUAAUAGCCUCTT
*Emilin-1-c*	Forward Reverse	GUUUCAGCCUCUACACAGGUUTT AACCUGUGUAGAGGCUGAAACTT

### Treatment of hDPSCs by rhEMILIN-1

rhEMILIN-1 (Proteintech, Wuhan, China) was used to treat cell culture plates where the coating density was 0, 0.1, 0.5, 1 and 5 μg/cm^2^. Specifically, the well plates were uniformly covered with the corresponding rhEMILIN-1 solution in a volume of 0.1 mL/cm^2^. The well plates were then incubated in the cell incubator with saturated humidity and 5% CO_2_ at 37 °C for 2 h. The coating solution was removed and the hDPSCs were immediately inoculated into well plates coated with rhEMILIN-1 for subsequent experiments. Cells were cultured for 1, 6 and 24 h to observe cell morphology and adherence using a phase contrast microscope (Leica, Wetzlar, Germany) and photographs of the cells were taken. Then, three images from each group were randomly selected for quantitative analysis of cell adhesion using ImageJ software (NIH, Bethesda, MD, USA).

### Immunofluorescence (IF)

hDPSCs were inoculated in rhEMILIN-1-coated wells for 24 h and their EMILIN-1 and phalloidin-labelled immunofluorescence was examined. Briefly, cells were fixed in 4% PFA fix solution for 15 min, permeabilized with 0.3% Triton X-100 for 15 min, blocked with 5% bovine serum albumin for 1 h and then incubated with rabbit anti-EMILIN-1 (bs-14583R; Bioss Bio Inc, Beijing, China) diluted 1:150 at 4 °C overnight. Cells were incubated with fluorescent secondary antibody (Thermo Fisher Scientific, Waltham, MA, USA) for 1 h at room temperature. Next, the cells were stained with phalloidin (Yeasen Biotechnology (Shanghai) Co., Ltd., Shanghai, China) for 1 h, and then the nuclei were stained with DAPI (Beyotime, Shanghai, China) for 5 min. The results of IF were observed and recorded under a EVOS FL Auto Fully Automated Fluorescent Intelligent Imaging System (Thermo Fisher Scientific, Waltham, MA, USA).

### Cell Counting Kit-8 (CCK-8) assay

In *Emilin-1* knockdown experiments, hDPSCs were inoculated at 2 × 10^3^ cells/well into 96-well plates (Jet Bio, Guangzhou, China), and then hDPSCs were transfected with *Emilin-1* siRNA for 24 h, following the transfection protocol above. In experiments with exogenous addition of rhEMILIN-1, hDPSCs were inoculated at 2 × 10^3^ cells/well in 96-well plates coated with 1 μg/cm^2^ rhEMILIN-1. When 0, 3, 5 and 7 d of culture in GM, the medium was replaced with 100 µL the GM containing 10 µL CCK-8 reagent (Dojindo, Kumamoto, Japan), and then, the plates were placed in the incubator to protect from light and incubated for 2 h. The absorbance value of the reaction solution at 450 nm was detected by EnSpire Multimode Plate Reader (Perkin-Elmer, Waltham, MA, USA), then proliferation curve of hDPSCs was plotted according to the obtained absorbance value.

### Osteo/Odontogenic differentiation of hDPSCs

When hDPSCs reached about 80% proliferation in GM, the medium was replaced with the osteo/odontogenic induction medium (OM; the GM supplemented with 5 mM β-glycerophosphate (Sigma, St. Louis, MO, USA), 50 µg/mL ascorbic acid (Solarbio, Beijing, China) and 100 nM dexamethasone (Solarbio, Beijing, China)). The medium was changed every 3 days. To examine the early osteo/odontogenic differentiation capacity and expression of osteo/odonto-specific genes in hDPSCs, the cells were cultured in OM for 7 days. To assay the mineralization ability of hDPSCs, cells were cultured with OM for 21 days. In addition, hDPSCs were cultured in OM for 0, 1, 4, 7, 14 and 21 days to detect expression of osteo/odonto-specific proteins and EMILIN-1, respectively. In *Emilin-1* knockdown experiments, transfected hDPSCs were cultured in OM for 7 days to examine the effect of *Emilin-1* knockdown on the osteo/odontogenic differentiation of hDPSCs. In experiments with exogenous addition of rhEMILIN-1, hDPSCs were inoculated into rhEMILIN-1-treated well plates and cultured in OM for 7 and 21 days, respectively.

### Alkaline phosphatase (ALP) staining

The hDPSCs were cultured in OM for 7 days. Following three PBS washes, hDPSCs were fixed in 4% PFA for 15 min. According to the instructions, ALP staining was done for 10 min with the BCIP/NBT ALP Color Development Kit (Beyotime, Shanghai, China). Pictures were obtained by using the Epson Perfection V330 scanner and the inverted light microscope.

### Alizarin red S (ARS) staining

The hDPSCs were cultured in OM for 21 days. Following three PBS washes, hDPSCs were fixed in 4% PFA for 15 min. The hDPSCs were then gently rinsed with distilled water to remove residual fixative and stained with 1% alizarin red S solution (Sigma, St. Louis, MO, USA) for 10 min. Images were acquired in the same methods as ALP staining. In addition, the stained hDPSCs were dissolved in 10% cetylpyridinium chloride and then the absorbance of this solution at 562 nm was measured using the EnSpire Multimode Plate Reader to quantify the ARS staining.

### Quantitative real-time PCR (qPCR)

The total RNA of the cells treated with different treatments was extracted by RNAiso Plus (TaKaRa, Tokyo, Japan). Then, the PrimeScript™ RT reagent kit (TaKaRa, Tokyo, Japan) was used for reverse transcription. qPCR was performed using QuantiNova SYBR Green PCR Kit (QIAGEN, Duesseldorf, Germany) in a fluorescence quantitative PCR instrument system (Bio-Rad, Hercules, CA, USA) at 95 °C for 2 min, and 40 cycles at 95 °C for 5 s and 60 °C for 10 s. *Gapdh* was regarded as an internal reference. Relative genes expression in this study were calculated through the 2 ^−ΔΔCt^ method. All target gene sequences are shown in Table 2.

**Table 2:** Sense and antisense primers for qPCR

*Genes*	Primers	Sequences (5'-3')
*Alp*	Forward Reverse	TAAGGACATCGCCTACCAGCTC TCTTCCAGGTGTCAACGAGGT
*Runx2*	Forward Reverse	CTTTACTTACACCCCGCCAGTC AGAGATATGGAGTGCTGCTGGTC
*Osx*	Forward Reverse	CCTCTGCGGGACTCAACAAC AGCCCATTAGTGCTTGTAAAGG
*Ocn*	Forward Reverse	CTCACACTCCTCGCCCTATTG CTCCCAGCCATTGATACAGGTAG
*Emilin-1*	Forward Reverse	CAGCCTCTACACAGGTTCCAG CACGTAGGCACACCAGTTCC
*Gapdh*	Forward Reverse	CTTTGGTATCGTGGA AGGACTC GTAGAGGCAGGGATGATGTTCT

*Abbreviations*: *Alp* alkaline phosphatase, *Runx2* runt-related transcription factor 2, *Osx* osterix, *Ocn* osteocalcin, *Gapdh* glyceraldehyde 3-phosphate dehydrogenase

### Western blot analysis

The hDPSCs were repeatedly washed with cold PBS and fully reacted with RIPA lysis buffer (Beyotime, Shanghai, China) containing the protease inhibitor phenylmethanesulfonyl fluoride (Beyotime, Shanghai, China) for 10 min. The cells were then scraped off and collected in a pre-cooled centrifuge tube. Next, the total extracted proteins were normalized using BCA protein analysis kit (Beyotime, Shanghai, China). The protein samples were separated using gels configured with TGX Stain-Free™ FastCast™ Acrylamide Solutions (Bio-Rad, Hercules, CA, USA). Subsequently, the protein was transferred from the gels to polyvinylidene fluoride membranes (Merck Millipore, Billerica, MA, USA) by a semi-dry transfer system (Bio-Rad, Hercules, CA, USA). After being blocked with 5% skim milk (Beyotime, Shanghai, China) for 2 h at room temperature, the membranes were incubated with primary antibodies overnight at 4 °C on a shaker. The membranes were then incubated with anti-mouse/rabbit IgG secondary antibodies conjugated with horseradish peroxidase (Bio-Rad, Hercules, CA, USA) for 2 h at room temperature. Secondary antibodies were diluted 1:3000 in 1% skim milk in TBST. The bands were incubated with BeyoECL Star chemiluminescence detection reagent (Beyotime, Shanghai, China) and visualized using Western blot analysis imaging system (Bio-Rad, Hercules, CA, USA). The primary antibodies used in this experiment were as follows: mouse anti-COL1 (ab6308; Abcam, Cambridge, MA, USA) diluted 1:1000; mouse anti-DMP1 (sc-81249; Santa Cruz Biotechnology, Santa Cruz, CA, USA) diluted 1:500; mouse anti-DSPP (sc-73632; Santa Cruz Biotechnology, Santa Cruz, CA, USA) diluted 1:500; rabbit anti-EMILIN-1(ab185953; Abcam, Cambridge, MA, USA) diluted 1:5000; rabbit anti-Runx2 (D1L7F; Cell Signaling Technology, Danvers, MA, USA) diluted 1:1000; mouse anti-OPN (sc-21742; Santa Cruz Biotechnology, Santa Cruz, CA, USA) diluted 1:500 and mouse anti-GAPDH (200,306-7E4; Zen-bio, Chengdu, China) diluted 1:5000. Among them, GAPDH was regarded as internal reference.

### Statistical analysis

Experiments were independently repeated at least in triplicate. The data were expressed by means ± standard deviation (SD). Statistical analysis was performed using GraphPad Prism 9 software (GraphPad, San Diego, CA). Comparison methods for significance of difference included student T test or one-way ANOVA, and P < 0.05 was considered statistically significant.

## Results

### Isolation, culture and identification of hDPSCs

**Fig. 1:**
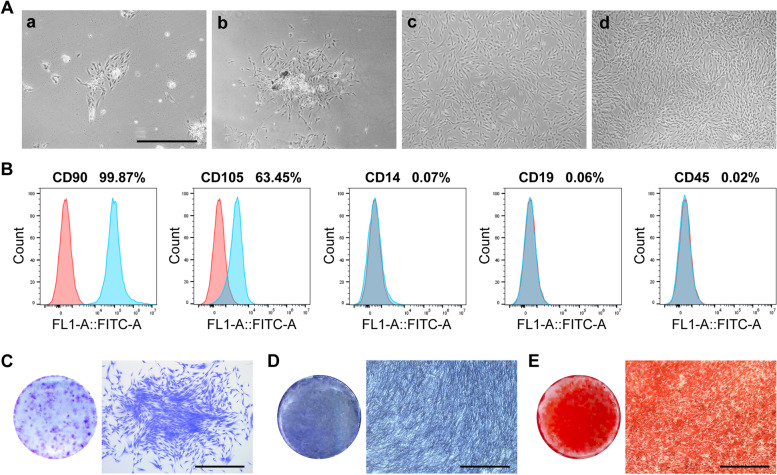
The isolation and characterization of hDPSCs. **A** Morphology of the human pulp stem cells (hDPSCs) colonies formed after 3 d (**a**) and 5 d (**b**) of pulp tissue isolation and culture. The fifth generation hDPSCs were cultured for 24 h (**c**), with cells arranged in bundles or spirals, and after 48 h (**d**), cells proliferated reached 80% confluence. **B** Flow cytometry to identify mesenchymal stem cell properties of hDPSCs. **C** Colony forming unit of hDPSCs. **D** Alkaline phosphatase (ALP) staining. **E** Alizarin red S (ARS) staining. Scale bars = 250 μm

The hDPSCs are typical fibroblast-like morphology of adherent cells. Under the microscope, individual cells are long spindle-shaped and arranged in bundles or spirals (Fig. 1A). hDPSCs are isolated and cultured, then, the cells can be stably passaged (Fig. 1Ac, Ad). Flow cytometry detected that the mesenchymal stem cell markers CD90 (99.87%) and CD105 (63.45%) were highly expressed in hDPSCs, while monocyte marker CD14 (0.07%), B cell marker CD19 (0.06%) and hematopoietic marker CD45 (0.02%) were all negative (Fig. 1B). When cultured at low density, hDPSCs formed cell colonies (Fig. 1C). hDPSCs have osteo/odontogenic differentiation potential [18]. ALP staining of hDPSCs was blue-purple when the cells were cultured in OM for 7 days (Fig. 1D). After 21 days of OM culture, ARS staining image of hDPSCs were red, and a large number of mineralized nodules could be observed under the microscope (Fig. 1E).

### EMILIN-1 expression increased during the osteo/odontogenic differentiation of hDPSCs in vitro

**Fig. 2:**
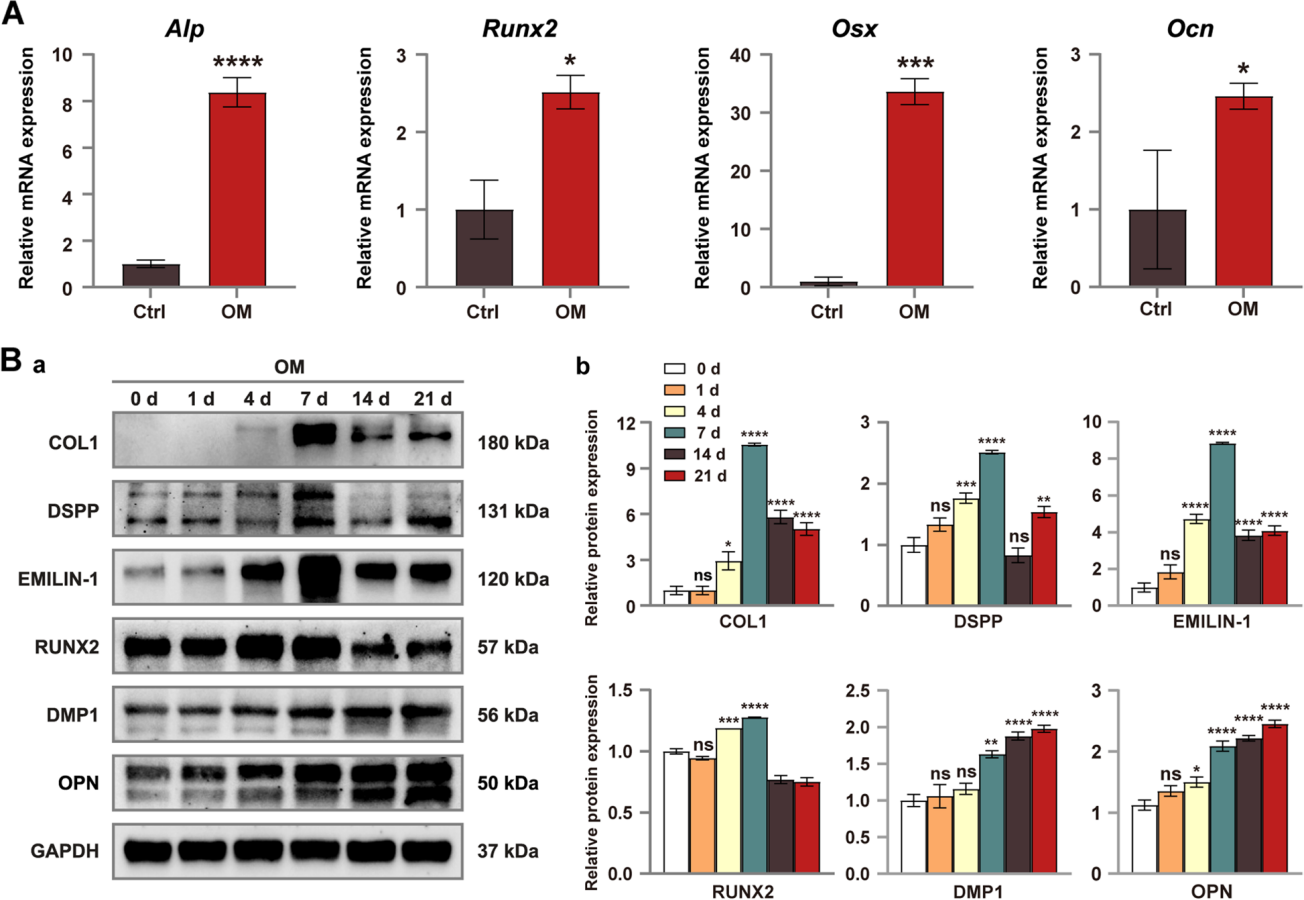
Increased expression of EMILIN-1 during osteo/odontogenic differentiation of hDPSCs. **A** Osteo/Odontogenic induction culture increased the mRNA expression levels of *Alp*, *Runx2*, *Osx*, and *Ocn* genes in hDPSCs. **B** The induction increased the expression levels of EMILIN-1 and osteo/odonto-specific proteins (**a**). Grayscale analysis of protein bands (**b**). Values are presented as mean ± standard deviation (SD). *P < 0.05; **P < 0.01; ***P < 0.001; ****P < 0.0001. The blots in **Ba** were cropped

The hDPSCs were grown in OM for 0, 1, 4, 7, 14, and 21 days. The Western bolt results showed that EMILIN-1 protein levels increased with the duration of induction time and reached a maximum on day 7, then decreased but remained at high expression levels (Fig. 2Ba, Bb; Fig. S1). After hDPSCs were cultured in OM for 7 days, the relative mRNA expression levels of their osteo/odonto-specific genes (*Alp*, *Runx2*, *Osx* and *Ocn*) were increased (Fig. 2A). Similarly, the relative expression levels of osteo/odonto-specific proteins (COL1, RUNX2, OPN, DSPP and DMP1) in hDPSCs increased with the prolonged osteo/odontogenic induction time. Specifically, the expression of COL1, RUNX2 and DSPP peaked at day 7 of induction and then began to decline (Fig. 2Ba, Bb; Fig. S1).

### *Emilin-1* knockdown inhibited osteo/odontogenic differentiation of hDPSCs

**Fig. 3:**
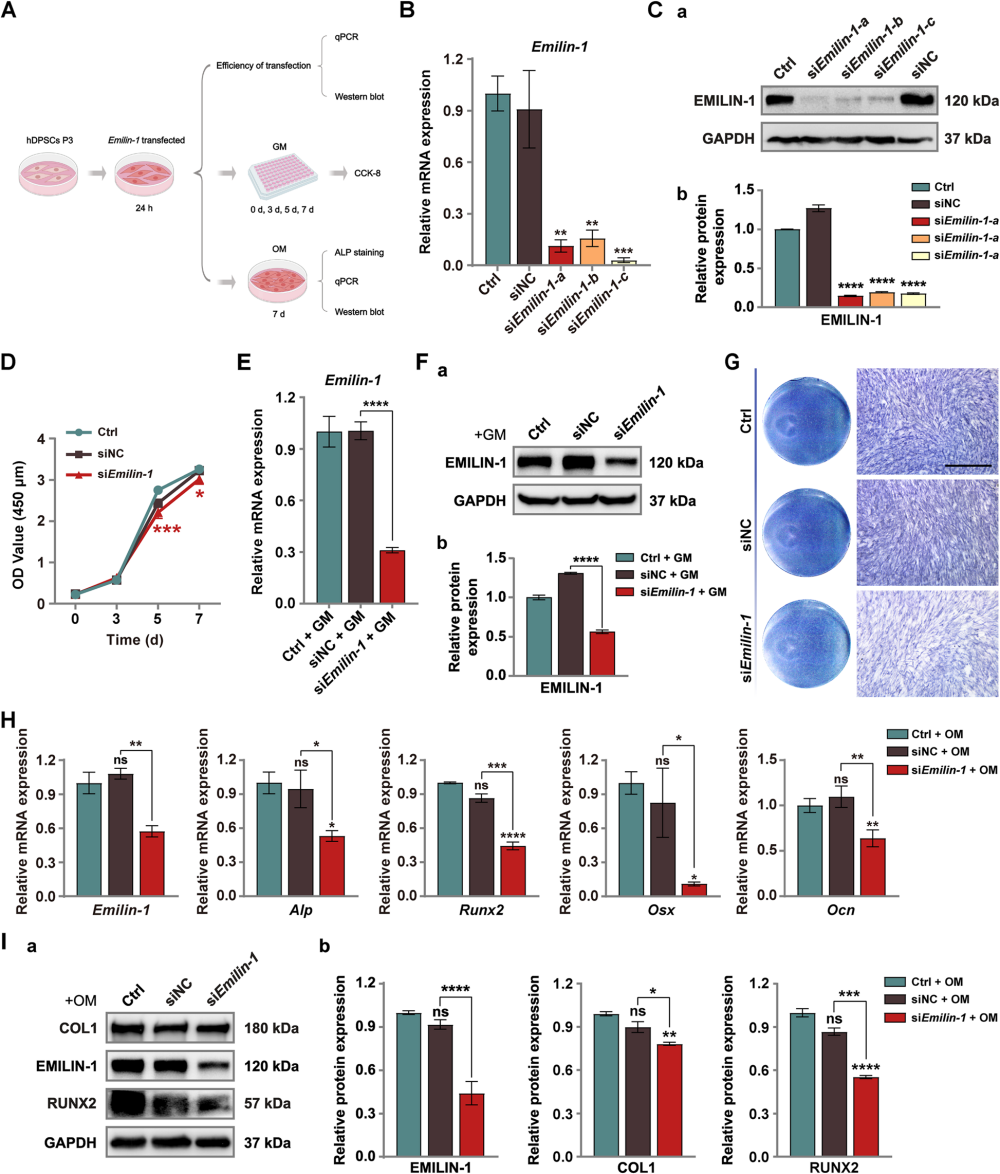
Knockdown of *Emilin-1* inhibited osteo/odontogenic differentiation of hDPSCs. **A** The summary diagram of the experimental workflow. **B** *Emilin-1* siRNA knockdown efficiency was confirmed by qPCR and **C** Western blot analysis (**a**). Grayscale analysis of protein bands (**b**). **D** *Emilin-1* knockdown resulted in decreased proliferation of hDPSCs detected by the Cell Counting Kit-8 (CCK-8) assay. **E** Good knockdown efficiency was maintained after 7 days of culture in growth medium as detected by qPCR and **F** Western blot analysis (**a**). Gray-scale analysis of protein bands (**b**). **G** ALP staining showed that *Emilin-1* knockdown decreased ALP activity in hDPSCs. Scale bars = 250 μm. **H** qPCR and **I** Western blot detected that the expression of EMILIN-1 and osteo/odonto-specific genes/proteins was reduced in transfected hDPSCs after 7 days of culture in osteo/odontogenic induction medium (**a**). Grayscale analysis of protein bands (**b**). Values are presented as mean ± standard deviation (SD). *P < 0.05; **P < 0.01; ***P < 0.001; ****P < 0.0001. The blots in **Ca**, **Fa**, **Ia** were cropped

To further validate the role of EMILIN-1 in the osteo/odontogenic differentiation of hDPSCs, we designed three gene sequences (*Emilin-1-a*, *Emilin-1-b* and *Emilin-1-c*) to knock down *Emilin-1* in hDPSCs and drew a summary diagram of the experimental workflow (Fig. 3A). hDPSCs were transfected with siRNA for 24 h, and the optimal interference sequence was screened by qPCR and Western blot analysis. Combining the two results, the knockdown rate of endogenous *Emilin*-1 was more than 70%, and the knockdown effect of *Emilin-1-a* was the best (Fig. 3B, Ca, Cb; Fig. S2A). Therefore, in this study, *Emilin-1-a* sequence was selected to interfere with *Emilin-1* in hDPSCs. The CCK-8 assay showed that the knockdown group had reduced cell proliferation at days 5 and 7 compared to the control group (Fig. 3D). In addition, the knockdown rate of transfected hDPSCs remained over 50% after 7 d of culture in GM (Fig. 3E, Fa, Fb; Fig. S2B). To evaluate the effect of *Emilin-1* knockdown on the osteo/odontogenic differentiation potential of hDPSCs, hDPSCs with *Emilin-1* knockdown were cultured in OM for 7 days. qPCR and Western blot analysis showed that the transfected hDPSCs still maintained a good knockdown effect after 7 days of culture in OM (Fig. 3H, Ia, Ib; Fig. S2C). Knockdown of endogenous *Emilin-1* attenuated the osteo/odontogenic differentiation ability of hDPSCs, as demonstrated by ALP staining (Fig. 3G). The relative mRNA expression levels of osteo/odonto-specific genes (*Alp*, *Runx2*, *Ocn*, *Osx*) were decreased in *Emilin-1* knockdown hDPSCs (Fig. 3H), which was consistent with the result of impaired ALP staining. In addition, the expression of osteo/odonto-specific proteins COL1 and RUNX2 was also decreased in hDPSCs after endogenous *Emilin-1* knockdown (Fig. 3Ia, Ib; Fig. S2C).

### rhEMILIN-1 promoted the adhesion of hDPSCs

**Fig. 4:**
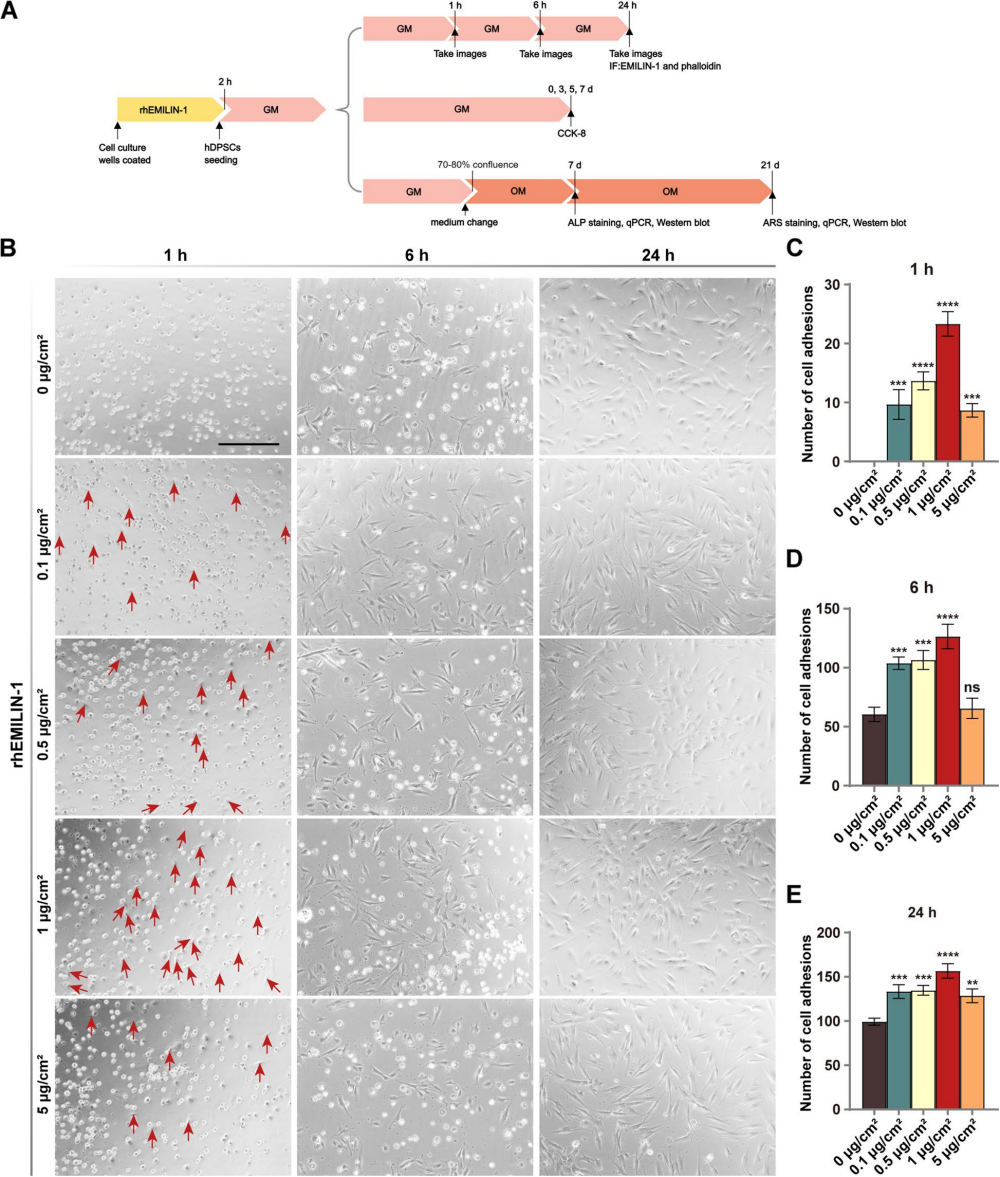
rhEMILIN-1 coating stimulated adhesion of hDPSCs. **A** The summary diagram of the experimental workflow. **B** Micrographs of hDPSCs seeded into rhEMILIN-1-coated wells at 1 h, 6 h and 24 h. The adhesion numbers of hDPSCs at **C** 1 h, **D** 6 h and **E** 24 h. The red arrows labeled the adherent cells at 1 h. Scale bars = 100 μm. Values are presented as mean ± standard deviation (SD). **P < 0.01; ***P < 0.001; ****P < 0.0001

**Fig. 5:**
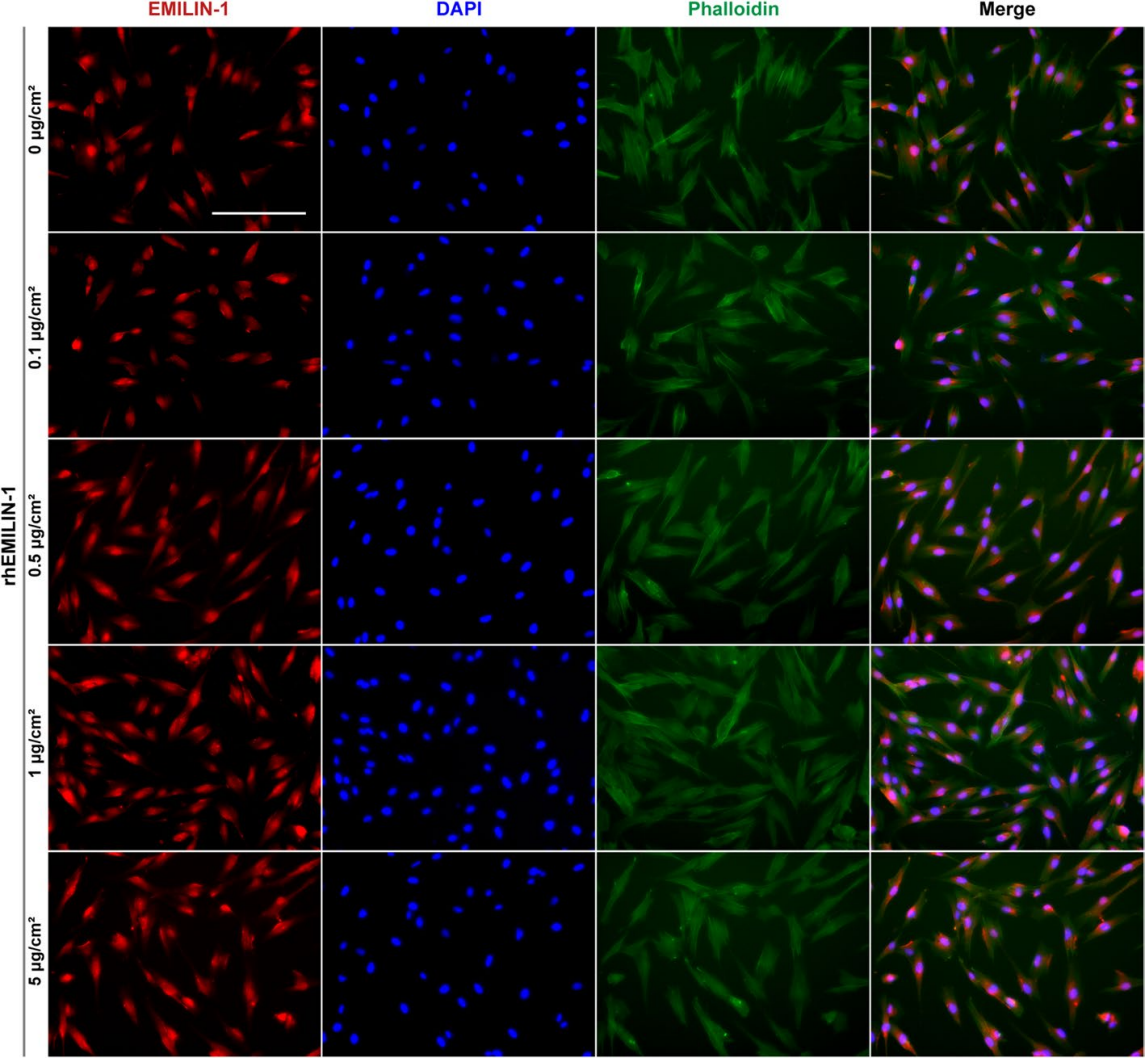
The rhEMILIN-1 coating had no effect on the morphology of hDPSCs. The representative images of EMILIN-1 and phalloidin-labeled immunofluorescence of hDPSCs when the cells were seeded in the coated wells for 24 h. Scale bars = 200 μm

A series of experiments were designed to detect the effect of rhEMILIN-1 on hDPSCs (Fig. 4A). To observe the effect of EMILIN-1 on cell adhesion, rhEMILIN-1 was coated onto wells at concentrations of 0, 0.1, 0.5, 1 and 5 µg/cm^2^. The results showed that some cells in the rhEMILIN-1-treated group started to adhere and form protrusions at 1 h, especially in the 1 µg/cm^2^ group, while almost all cells in the control group remained spherical (Fig. 4B, C). At 6 and 24 h, the adherent cells in each group were shuttle-shaped and flattened, and the number of cell adhesions was higher in the rhEMILIN-1-treated group compared with the control group, especially in the 1 µg/cm^2^ group (Fig. 4B, D, E). Therefore, 1 µg/cm^2^ rhEMILIN-1 was selected for subsequent experiments. Immunofluorescence staining for EMILIN-1 and phalloidin was performed on hDPSCs at 24 h of culture. The cell morphology of hDPSCs cultured on wells coated with different concentrations of rhEMILIN-1 were all shuttle-shaped and their morphology was similar to that of the control group (Fig. 5). In addition, EMILIN-1 was expressed in the nucleus and cytoplasm of hDPSCs and did not differ significantly among groups (Fig. 5).

### rhEMILIN-1 promoted osteo/odontogenic differentiation of hDPSCs

**Fig. 6:**
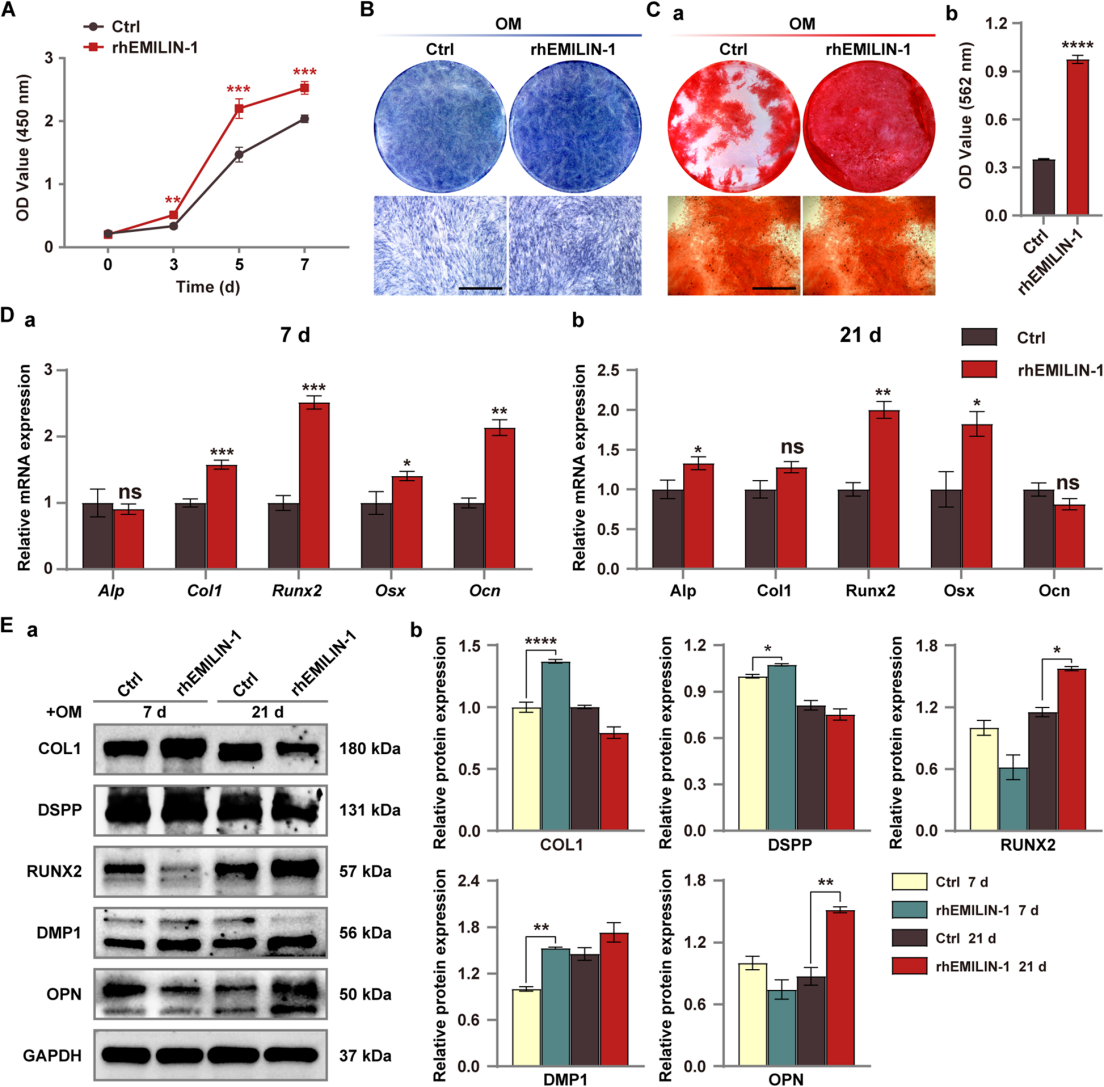
rhEMILIN-1 coating stimulated osteo/odontogenic differentiation of hDPSCs. **A** CCK8 assay showed that rhEMILIN-1 treatment significantly increased the proliferation of hDPSCs. **B** ALP staining (7 days) showed that rhEMILIN-1 decreased ALP activity in hDPSCs. Scale bars = 250 μm. **C** ARS staining (21 days) showed that rhEMILIN-1 treatment significantly increased the mineralization capacity of hDPSCs (**a**). Mineralized nodules was quantified (**b**). Scale bars = 250 μm. **D** qPCR detected that rhEMILIN-1 treatment increased the relative mRNA expression of osteo/odonto-specific genes at osteo/odontogenesis day 7 (**a**) and osteo/odontogenesis day 21(**b**). **E** Western blot detected that rhEMILIN-1 treatment increased the expression of osteo/odonto-specific proteins in hDPSCs at early and late stages of induction (**a**). Grayscale analysis of protein bands (**b**). Values are presented as mean ± standard deviation (SD). *P < 0.05; **P < 0.01; ***P < 0.001; ****P < 0.0001. The blots in **Ea** were cropped

The bottom of the well plates was treated with rhEMILIN-1 to examine the effect of EMILIN-1 on proliferation and osteo/odontogenic differentiation of hDPSCs. CCK-8 assay revealed that rhEMILIN-1 coating promoted the proliferation of hDPSCs (Fig. 6A). For osteo/odontogenic differentiation, rhEMILIN-1-treated hDPSCs were cultured for 7 or 21 days in OM. The results showed a significant increase in the intensity of ALP staining (7 d) and calcium deposition in ARS staining (21 d) in the rhEMILIN -1-treated group (Fig. 6B, Ca, Cb). The expression mRNA levels of osteo/odonto-specific markers (*Alp*, *Col1*, *Runx2*, *Osx* and *Ocn*) were measured on day 7 and day 21 respectively. Expression of *Col1*, *Runx2*, *Osx* and *Ocn* were significantly upregulated at day 7 (Fig. 6Da), while *Alp*, *Runx2*, and *Osx* were upregulated at day 21 (Fig. 6Db). In addition, we also assessed the expression levels of specific proteins involved in osteo/odontogenic differentiation. rhEMILIN-1 treatment increased the protein expression levels of COL1, DSPP and DMP1 in hDPSCs at day 7 of induction, while RUNX2 and OPN expression levels also increased at day 21(Fig. 6Ea, Eb; Fig. S3).

## Discussion

Osteo/Odontogenic differentiation of hDPSCs plays an important role in dentinogenesis, tooth and bone repair and regeneration [19], therefore, identification of molecules regulating the process will contribute to the study of tooth and bone regeneration, opening up further possibilities for addressing long-standing teeth and bones defects and related diseases. Recently, researchers have focused much attention on the role of ECM proteins in the cell survival microenvironment in the osteo/odontogenic differentiation of DPSCs, such as COL1, osteopontin (OPN), osteocalcin (OCN) and fibronectin [20, 21]. Our previous studies found for the first time that the existence of EMILIN-1 in human dentin [16]. In the present study, we found for the first time that EMILIN-1 expression increased with the duration of induction of osteo/odontogenic differentiation of hDPSCs in vitro. Furthermore, silencing of endogenous EMILIN-1 and addition of exogenous rhEMILIN-1 inhibited and increased osteo/odontogenic differentiation of hDPSCs, respectively. These findings suggested that EMILIN-1 may play a facilitating role in the osteo/odontogenic differentiation of hDPSCs.

In this study, we extracted hDPSCs with a typical fibroblast-like morphology, which expressed typical surface markers of human MSCs and showed strong proliferation and osteo/odontogenic differentiation potential. Based on the above findings and a series of reports on the identification of hDPSCs [1, 22], it can be concluded that the cells we extracted are indeed human MSCs. In this study, osteo/odonto-specific genes/proteins expression levels of ALP, COL1, RUNX2, OSX and DSPP [23, 24] were significantly upregulated in hDPSCs during the early stages of osteo/odontogenic induction, with a peak at day 7. The expression of DMP1, OCN and OPN were also up-regulated until late induction phase. Most importantly, the protein level expression of EMILIN-1 was significantly up-regulated during induction, reaching a maximum at day 7, and was also highly expressed in the late induction phase. Combining these results, we propose the hypothesis that EMILIN-1 may regulate the osteo/odontogenic differentiation of hDPSCs.

To validate the hypothesis, we knocked down *Emilin-1* in hDPSCs using siRNA. The results indicated that *Emilin-1* knockdown significantly impaired the proliferation and osteo/odontogenic differentiation of hDPSCs. This is consisted with a speculation proposed by Thomas Imhof et al. that EMILIN-1 may be involved in odontoblasts differentiation, dentinogenesis, and secondary dentin matrix formation [25]. Several studies have shown that EMILINs are important factors in osteo/odontogenesis. Proteomic analysis of mouse femoral cartilage discovered substantial expression of EMILIN-1 in the cartilage matrix [26]. EMILIN-2 was involved in the regulation of the differentiation of MSCs and hematopoietic progenitor cells by being deposited in the extracellular matrix of the bone marrow in an age-dependent manner [27]. EMILIN-3 was highly expressed in osteogenic mesenchyme [28]. Thus, these findings of these studies combined with our findings provide further evidence that EMILIN-1 may play a positive role in the osteo/odontogenic differentiation of hDPSCs.

Next, to further verify the effects of EMILIN-1 on the proliferation and osteo/odontogenic differentiation of hDPSCs, we treated hDPSCs with rhEMILIN-1. It was found that EMILIN-1 stimulated the adhesion and growth of hDPSCs and also enhanced ALP activity and mineralized nodules formation, as well as the expression of osteo/odonto-specific genes/proteins. Similarly, Smitha Mathews et al. found that culturing BMSCs in plates treated with ECM proteins (COL1, fibronectin, laminin and vitronectin) altered the cell growth patterns and induce osteogenic differentiation of hBMSCs [29]. It has been reported that DMP1 promoted adhesion and proliferation of human MSCs, as well as osteogenic differentiation and mineralized matrix formation when the DMP1 coatings were applied to human MSCs [30]. In addition, the laminin coating contributes to the odontogenic differentiation of hDPSCs and the formation of odontoblast layer in pulp regeneration [31]. Taken together, these data indicated that rhEMILIN-1 could promote proliferation and osteo/odontogenic differentiation of hDPSCs.

It is well known that Hedgehog (Hh), Wnt and TGF-β signaling play important roles in promoting osteo/odontogenic differentiation of MSCs [32]. Studies show that EMILINs can regulate the bioavailability of Hedgehog and Wnt ligands [33]. EMILIN- 3 regulated the effectiveness of Hh ligands by interacting with the permissive factor Scube2 in the notochord sheath [34]. EMILIN-2 can interact directly with Wnt1 ligands to affect the activation of Wnt signaling [35]. However, it was shown that EMILIN-1 inhibits TGF-β signaling, for example, EMILIN-1 blocked the potential activation of TGF-β outside the cell by binding to immature pro-TGF-β, which halted further processing of pro-TGF-β [36]. In addition, EMILIN-1 interfered with the binding of TGF-β ligands to their cognate receptors [37]. However, this regulatory mechanism may currently be limited to the vascular system in vivo [36–39], and whether EMILIN-1 inhibits TGF-β signaling in bone metabolism has not been reported so far. Therefore, more studies are needed to elucidate the interaction between EMILIN-1 and TGF-β signaling in bone metabolism [36–39]. Based on the above information, we hypothesize that EMILIN-1 may be involved in Hh, Wnt and/or TGF-β signaling pathways to regulate the osteo/odontogenic differentiation of hDPSCs, but more experiments are needed to confirm these hypotheses.

The present study explored for the first time the effect of EMILIN-1 on osteo/odontogenic differentiation of hDPSCs. However, there are some limitations here. First, the effect of overexpression EMILIN-1 in hDPSCs needs to be evaluated in future. Second, the effects of EMILIN-1 on regenerative repair of teeth and bone tissue needs to be tested. Third, the mechanism of EMILIN-1 in promoting osteo/odontogenic differentiation of hDPSCs requires more experiments to confirm. Nevertheless, this study verified the role of EMILIN-1 in the osteo/odontogenic differentiation of hDPSCs, providing additional possibilities for the study of tooth and bone regeneration and the treatment of tooth and bone defects.

## Conclusion

Taken together, our study demonstrates that EMILIN-1 plays a facilitative role in the proliferation and osteo/odontogenic differentiation of hDPSCs. This study will contribute to elucidate the osteo/dentinogenic differentiation process of hDPSCs and the dentinogenesis process, and provide new ideas for the regeneration of tooth and bone tissues as well as the treatment of related diseases.

## Supplementary Information

**Additional file 1. **All the original Western Blots in the article.

## Data Availability

The datasets generated or analyzed during this study are included in this published article and its supplementary information file. Other images are available from the corresponding author on reasonable request.

## Abbreviations

ALP: Alkaline phosphatase
α-MEM: Alpha-Minimum Essential Medium
ARS: Alizarin red S
CCK-8: Cell Counting Kit-8
COL1: Type I collagen
DMP1: Dentin matrix protein 1
DSPP: Dentin sialophosphoprotein
ECM: Extracellular matrix
EMILIN-1: Elastin microfibril interface-located protein-1
EMILINs: Elastin Microfibril Interface Located proteINs
FBS: Fetal bovine serum
FITC: Fluorescein isothiocyanate
GAPDH: Glyceraldehyde 3-phosphate dehydrogenase
GM: Growth medium
hBMSCs: Human bone marrow mesenchymal stem cells
hDPSCs: Human dental pulp stem cells
Hh: Hedgehog
hMSCs: Human mesenchymal stem cells
mRNA: Messenger ribonucleic acid
OCN: Osteocalcin
OM: Osteo/odontogenic induction medium
OPN: Osteopontin
OSX: Osterix
PBS: Phosphate buffered saline
PE: Phycoerythrin
PFA: Paraformaldehyde
qPCR: Quantitative real-time polymerase chain reaction
rhEMILIN-1: Recombinant human EMILIN-1 protein
RUNX2: Runt-related transcription factor 2
siRNA: Small interfering RNA
TGF-β: Transforming growth factor-β
