# Correction to: The role of EMILIN-1 in the osteo/odontogenic differentiation of dental pulp stem cells

**DOI:** 10.1186/s12903-025-05759-z

**Published:** 2025-03-21

**Authors:** Pingmeng Deng, Jing Huang, Qixuan Zhang, Yuejia Li, Jie Li

**Affiliations:** 1 College of Stomatology, Chongqing Medical University, 426# Songshibei Road, Yubei District, Chongqing, 401147 People’s Republic of China; 2 Chongqing Key Laboratory of Oral Diseases and Biomedical Sciences, Chongqing, People’s Republic of China; 3 Chongqing Municipal Key Laboratory of Oral Biomedical Engineering of Higher Education, Chongqing, People’s Republic of China

**Correction to: BMC Oral Health (2023) 23:203**

10.1186/s12903-023-02905-3

In this article [1], the representative microscopic image of the rhEMILIN-1 treated group in Fig. 6Ca was mistakenly duplicated from the control group image. The incorrect and corrected versions of Fig. 6 are displayed below.

Incorrect Fig. 6.

**Fig. 6:**
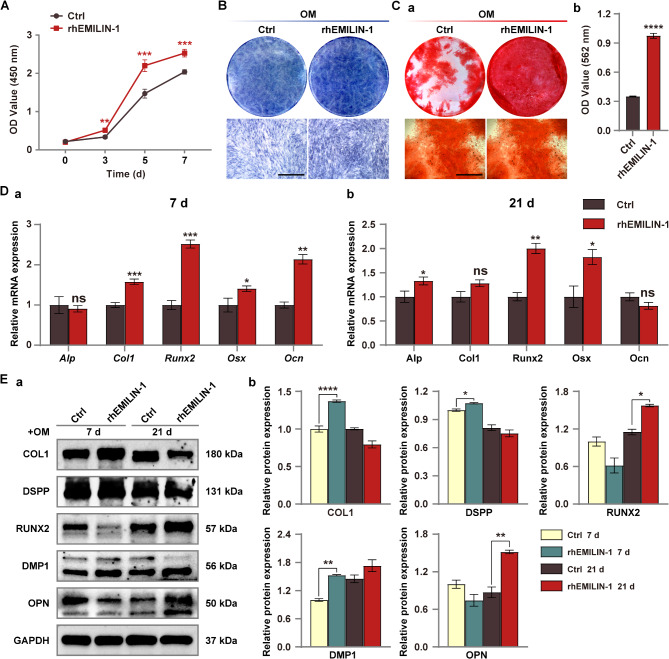
rhEMILIN-1 coating stimulated osteo/odontogenic differentiation of hDPSCs. **A** CCK8 assay showed that rhEMILIN-1 treatment significantly increased the proliferation of hDPSCs. **B** ALP staining (7 days) showed that rhEMILIN-1 decreased ALP activity in hDPSCs. Scale bars = 250 μm. **C** ARS staining (21 days) showed that rhEMILIN-1 treatment significantly increased the mineralization capacity of hDPSCs (**a**). Mineralized nodules was quantified (**b**). Scale bars = 250 μm. **D** qPCR detected that rhEMILIN-1 treatment increased the relative mRNA expression of osteo/odonto-specific genes at osteo/odontogenesis day 7 (**a**) and osteo/odontogenesis day 21(**b**). **E** Western blot detected that rhEMILIN-1 treatment increased the expression of osteo/odonto-specific proteins in hDPSCs at early and late stages of induction (**a**). Grayscale analysis of protein bands (**b**). Values are presented as mean ± standard deviation (SD). *P < 0.05; **P < 0.01; ***P < 0.001; ****P < 0.0001. The blots in **Ea** were cropped

Correct Fig. 6.

**Fig. 6:**
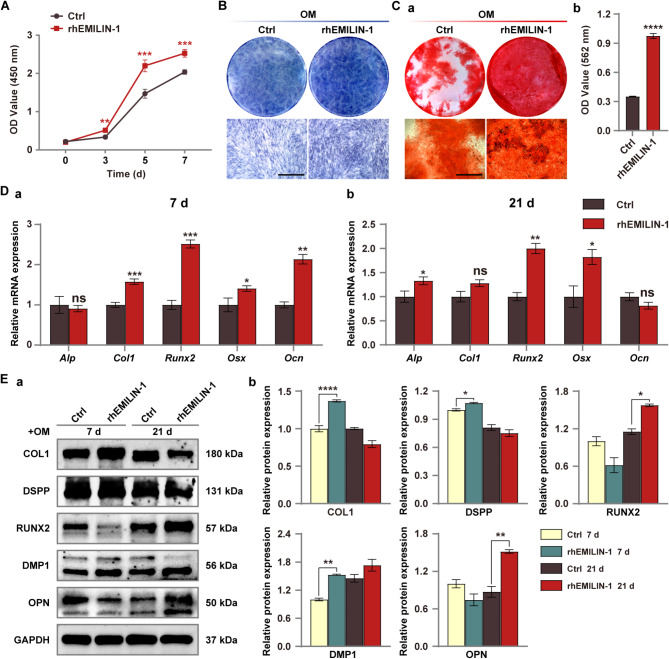
rhEMILIN-1 coating stimulated osteo/odontogenic differentiation of hDPSCs. **A** CCK8 assay showed that rhEMILIN-1 treatment significantly increased the proliferation of hDPSCs. **B** ALP staining (7 days) showed that rhEMILIN-1 decreased ALP activity in hDPSCs. Scale bars = 250 μm. **C** ARS staining (21 days) showed that rhEMILIN-1 treatment significantly increased the mineralization capacity of hDPSCs (**a**). Mineralized nodules was quantified (**b**). Scale bars = 250 μm. **D** qPCR detected that rhEMILIN-1 treatment increased the relative mRNA expression of osteo/odonto-specific genes at osteo/odontogenesis day 7 (**a**) and osteo/odontogenesis day 21(**b**). **E** Western blot detected that rhEMILIN-1 treatment increased the expression of osteo/odonto-specific proteins in hDPSCs at early and late stages of induction (**a**). Grayscale analysis of protein bands (**b**). Values are presented as mean ± standard deviation (SD). *P < 0.05; **P < 0.01; ***P < 0.001; ****P < 0.0001. The blots in **Ea** were cropped
