# Supplementary material for: The role of EMILIN-1 in the osteo/odontogenic differentiation of dental pulp stem cells
Source: BMC Oral Health. 2023 Apr 6;23:203. doi: 10.1186/s12903-023-02905-3 (PMC10077624; doi:10.1186/s12903-023-02905-3)
Supplement: Supplementary file 1 — Additional file 1. All the original Western Blots in the article. [file 12903_2023_2905_MOESM1_ESM.pdf]

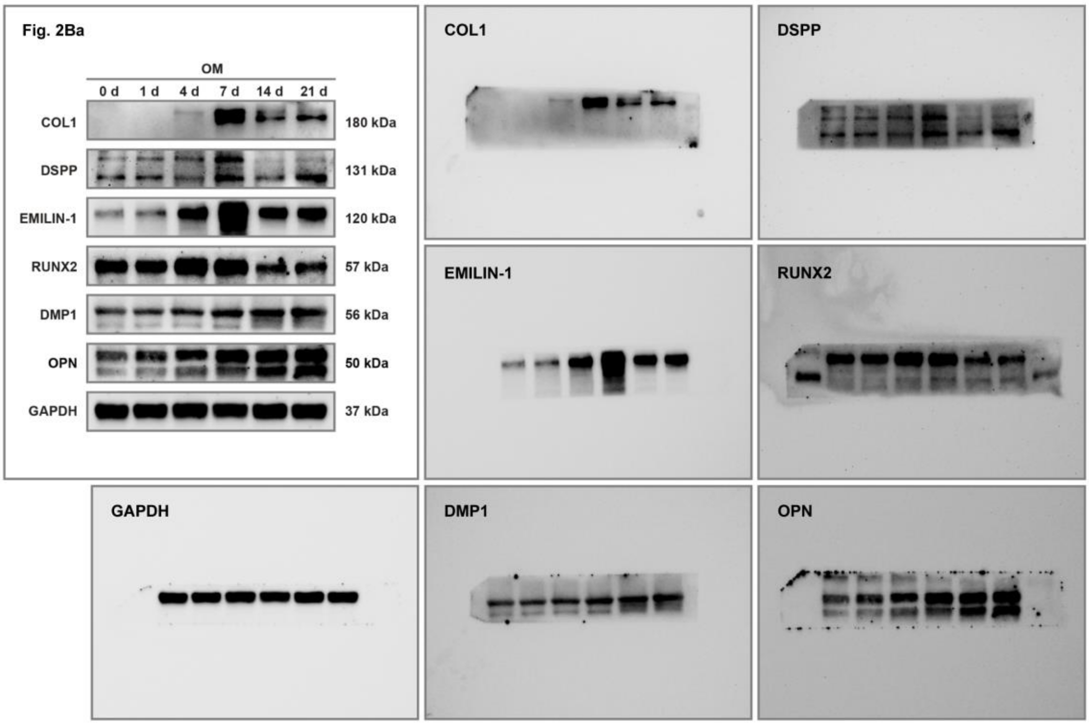

Fig. S1 Original images of Western blot for Fig. 2Ba. Protein levels of EMILIN-1 and osteo/odonto-specific proteins (COL1, DSPP, RUNX2, DMP1 and OPN) in hDPSCs at 0, 1, 4, 7, 14 and 21 days of osteo/odontogenic induction. GAPDH was selected as the internal control.

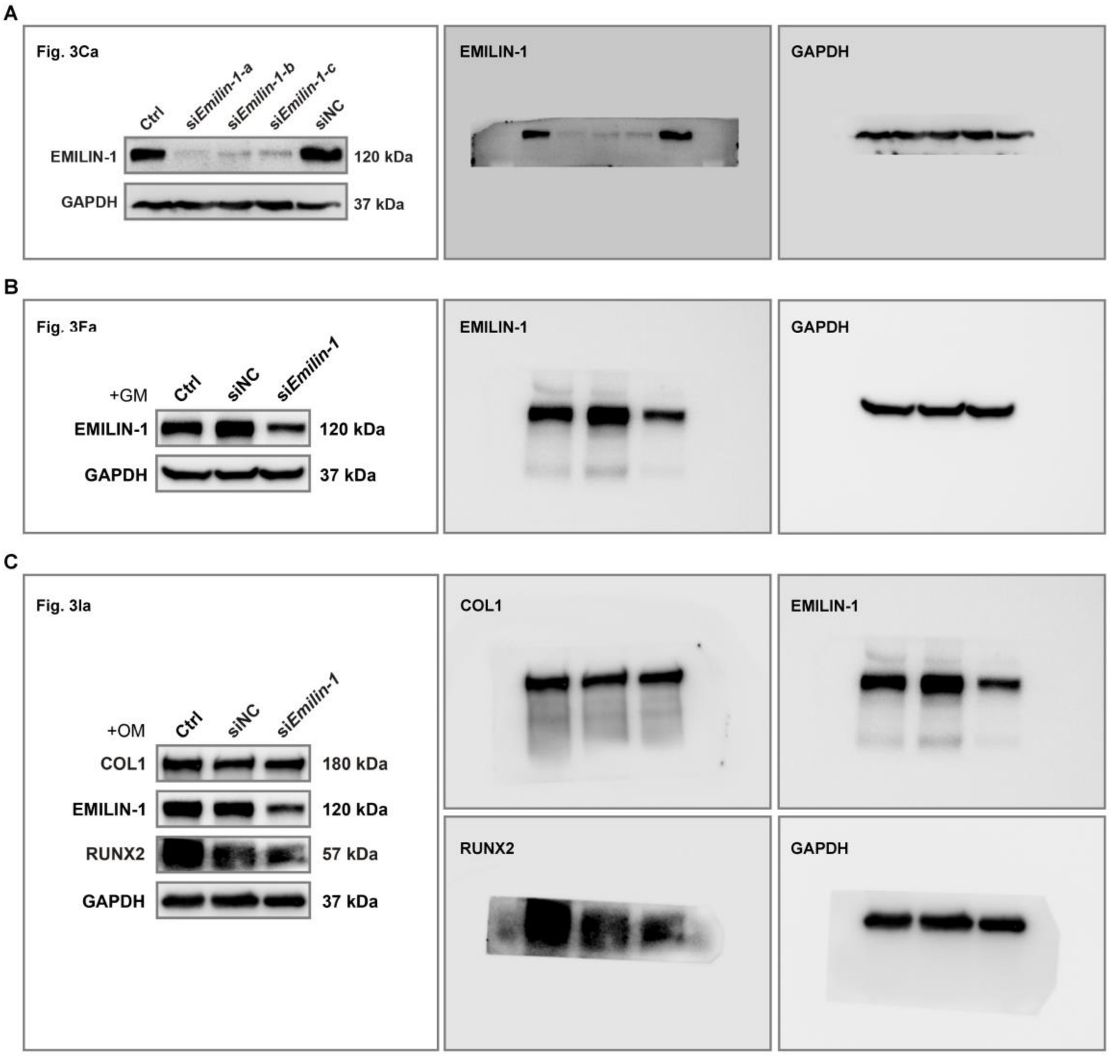

Fig. S2 Original images of Western blot for Fig.3. **A** Original images of Western blot for Fig. 3Ca. Protein levels of EMILIN-1 in hDPSCs transfected with siNC, si*EMILIN-1-a*, si*EMILIN-1-b* and si*EMILIN-1-c*. **B** Original images of Western blot for Fig. 3Fa. Protein levels of EMILIN-1 in transfected hDPSCs after 7 days of culture in growth medium. **C** Original images of Western blot for Fig. 3Ia. Protein levels of EMILIN-1 and osteo/odonto-specific proteins (COL1 and RUNX2) in transfected hDPSCs after 7 days of culture in osteo/odontogenic induction medium. GAPDH was selected as the internal control.

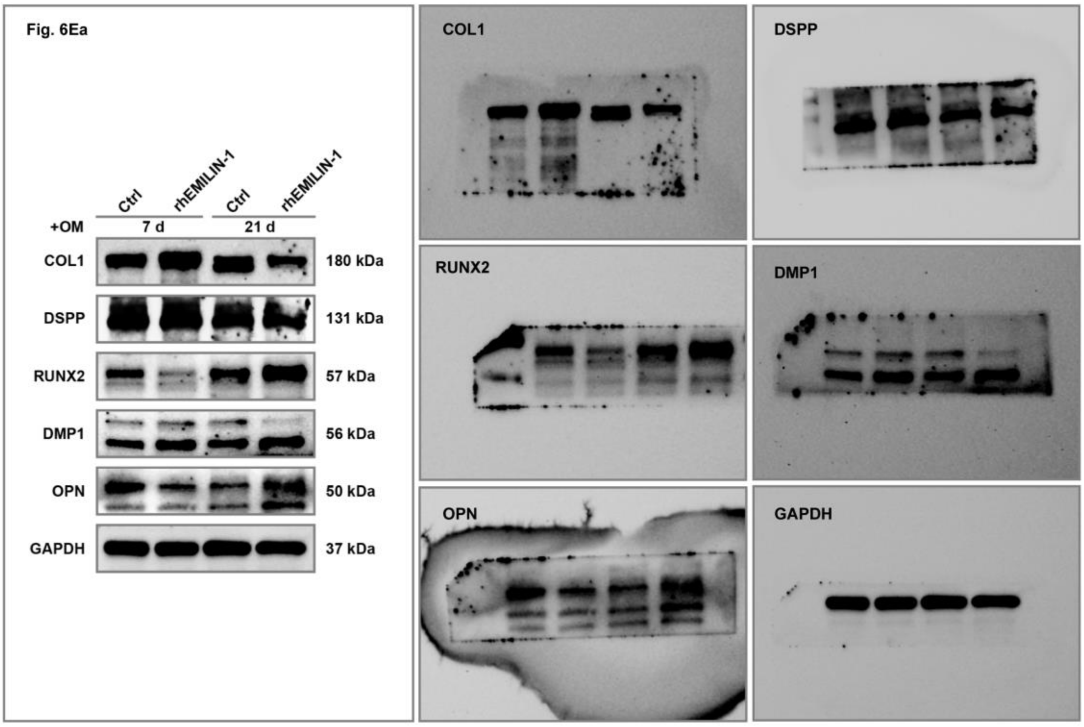

Fig. S3 Original images of Western blot for Fig. 6Ea. Protein levels of osteo/odonto-specific proteins (COL1, DSPP, RUNX2, DMP1 and OPN) in hDPSCs treated with rhEMILIN-1 for 7 and 21 days. GAPDH was selected as the internal control.
